# Multimodal prehabilitation program for patients undergoing elective surgery for colorectal cancer: a scoping review

**DOI:** 10.3389/fonc.2025.1532624

**Published:** 2025-04-30

**Authors:** Shilin Gao, Yuhua He, Lili Jiang, Jie Yang

**Affiliations:** Colorectal Cancer Center, West China Hospital, Sichuan University/West China School of Nursing, Sichuan University, Chengdu, Sichuan, China

**Keywords:** multimodal prehabilitation, preoperative, colorectal cancer, review, surgy

## Abstract

**Objectives:**

Multimodal prehabilitation has been widely used in patients undergoing surgery for colorectal cancer and has improved clinical outcomes. The aim of this scoping review is to review the content and current state of clinical practice of multimodal prehabilitation programs.

**Methods:**

A systematic literature review of multimodal prehabilitation studies in patients undergoing colorectal cancer surgery was conducted according to the PRISMA extension framework for scoping reviews. The literature was searched via the PubMed, Web of Science, SCOPUS, EMBASE and Cochrane Library databases. The results of the study included the components of multimodal prehabilitation (exercise, nutritional, and psychological interventions) and related evaluation indicators, duration, and compliance-related components.

**Results:**

This review included 12 studies with 9 randomized controlled trials, 1 pilot intervention study, 1 cohort study, and 1 mock-target trial design. Specific protocols for multimodal rehabilitation training vary widely, ranging in duration from 2–8 weeks, and were implemented in healthcare facilities, the community, and at home. Adherence rates ranged from 50% to almost 100%. Common outcome indicators include the 6-minute walk test, comorbidities, length of hospitalization, health-related quality of life, and several anxiety assessment scales.

**Conclusion:**

Current evidence suggests that multimodal preconditioning has a positive effect on the clinical prognosis of patients undergoing elective colorectal cancer surgery. However, owing to the heterogeneity of multimodal rehabilitation in terms of implementation protocols and evaluation metrics, many high-quality studies are still needed to explore the optimal model of multimodal rehabilitation and promote its standardization.

## 1 Introduction

Colorectal cancer (CRC) is the third most common cancer in the world and the second leading cause of cancer death globally, and is an important component of morbidity and mortality in the global population (1). The International Agency for Research on Cancer (IARC) reported (2) that in 2020 there were 1,932,000 new cases of CRC diagnosed globally, of which 930,000 will die. Unfortunately, 3.2 million new cases and 1.6 million deaths from CRC are expected annually by 2040, with the incidence increasing in younger people. Despite the fact that countries are actively promoting surgery-based comprehensive treatment modalities and have achieved some success, the morbidity and mortality rates of CRC remain high globally (3). The prevention and treatment experiences of some developed countries indicate that current CRC prevention strategies rely mainly on early screening and diagnosis, improvements in lifestyle and the environment, and polypectomy and aspirin administration, whereas CRC treatment modalities include surgery, chemotherapy, radiotherapy and immunotherapy (4). However, colorectal cancer surgical patients usually suffer from preoperative malnutrition, wasting and decreased fitness, and even cachexia and sarcopenia, all of which have been shown to be associated with poor clinical outcomes (5). In addition, surgical trauma triggers a series of metabolic changes in the body, including hormonal, hematologic, metabolic, and immunologic changes, a process known as surgical stress. The aim of prehabilitation is to optimize the various risk factors present in the patient during the preoperative waiting period and to improve the patient’s preoperative physiological and metabolic reserve to better withstand the trauma of surgery, reduce the incidence of perioperative complications and mortality, and optimize the patient’s clinical outcome (6).

Multimodal prehabilitation has been widely used in cancer patients, with initial preoperative rehabilitation focusing on a single exercise regimen to improve the patient’s functional reserve, followed by the addition of nutritional interventions to correct existing nutritional problems, and to help mitigate the patient’s perioperative trauma-related catabolic damage (7). Finally, psychological interventions were introduced to improve adherence to the treatment regimen while enhancing the patient’s intrinsic drive for exercise and nutritional interventions (8). Thus, a well-established multimodal prehabilitation program consists of three components: exercise, nutritional interventions, and psychological interventions, which can also be added to ameliorate poor lifestyles and anemia, among others. Despite the existence of a large body of data on multimodal prehabilitation programs, significant heterogeneity remains for each part of the intervention program, which has an important influence on further analysis.

Although the understanding of preoperative multimodal prehabilitation for rectal cancer is incomplete, the current growing body of clinical evidence gives us confidence in the future. This review will summarize the impact of multimodal prehabilitation on current clinical practice on the basis of the evidence for its plausibility and speculate on directions for further development.

## 2 Materials and methods

The PRISMA Extension Framework for Scoping Reviews (PRISMA-ScR) checklist was adopted in our study (9).

### 2.1 Search strategy

A comprehensive literature search of the PubMed, Web of Science, SCOPUS, EMBASE and Cochrane Library databases was conducted by two authors for studies published from the inception to August 18, 2024. The systematic search in Medical Subject Headings (MeSH) was conducted on the basis of the identified research questions to ensure the accuracy of the final search. The chosen key terms are: “Colorectal cancer”, and “Prehabilition”. The search strategy was customized basis of the characteristics of each database (**Supplementary Appendix 1** shows an example of the search strategy for the PubMed database). All findings were imported into EndNote (version X9.2) and duplicates were removed.

### 2.2 Eligibility criteria

The PICOTS model (10) is widely used in evidence-based healthcare as a guideline for developing search strategies and characterizing clinical studies. The following inclusion and exclusion criteria were developed for our study on the basis of the PICOTS model (**Table 1**).

**Table 1:** Eligibility criteria.

Items	Inclusion criteria	Exclusion criteria
Population	Adults with a clinical diagnosis of CRC	Not applicable
Interventions	Multimodal prehabilitation or one or two of them	Not applicable
Controls	Self before and after control or controls with other populations, etc.	Not applicable
Outcomes	Outcomes produced by an intervention by prehabilitation with a population	Not applicable
Time	Prehabilitation intervention duration	Not applicable
Study design	Various trial types published in English	Commentaries, Book reviews, Conference abstracts, etc.

Two authors performed an initial analysis of the study to minimize bias by screening the title and abstract independently, and a third author was responsible for resolving any discrepancies.

### 2.3 Data screening and extraction

Following the completion of the selection process, two authors extracted the characteristics of the studies including author, year, and country of origin, study design, participants, sample size, prehabilitation protocol and outcome measures, via a predesigned standardized data extraction form. In addition, one of the authors extracted outcome measures from the results and counted their frequency.

## 3 Results

**Figure 1:**
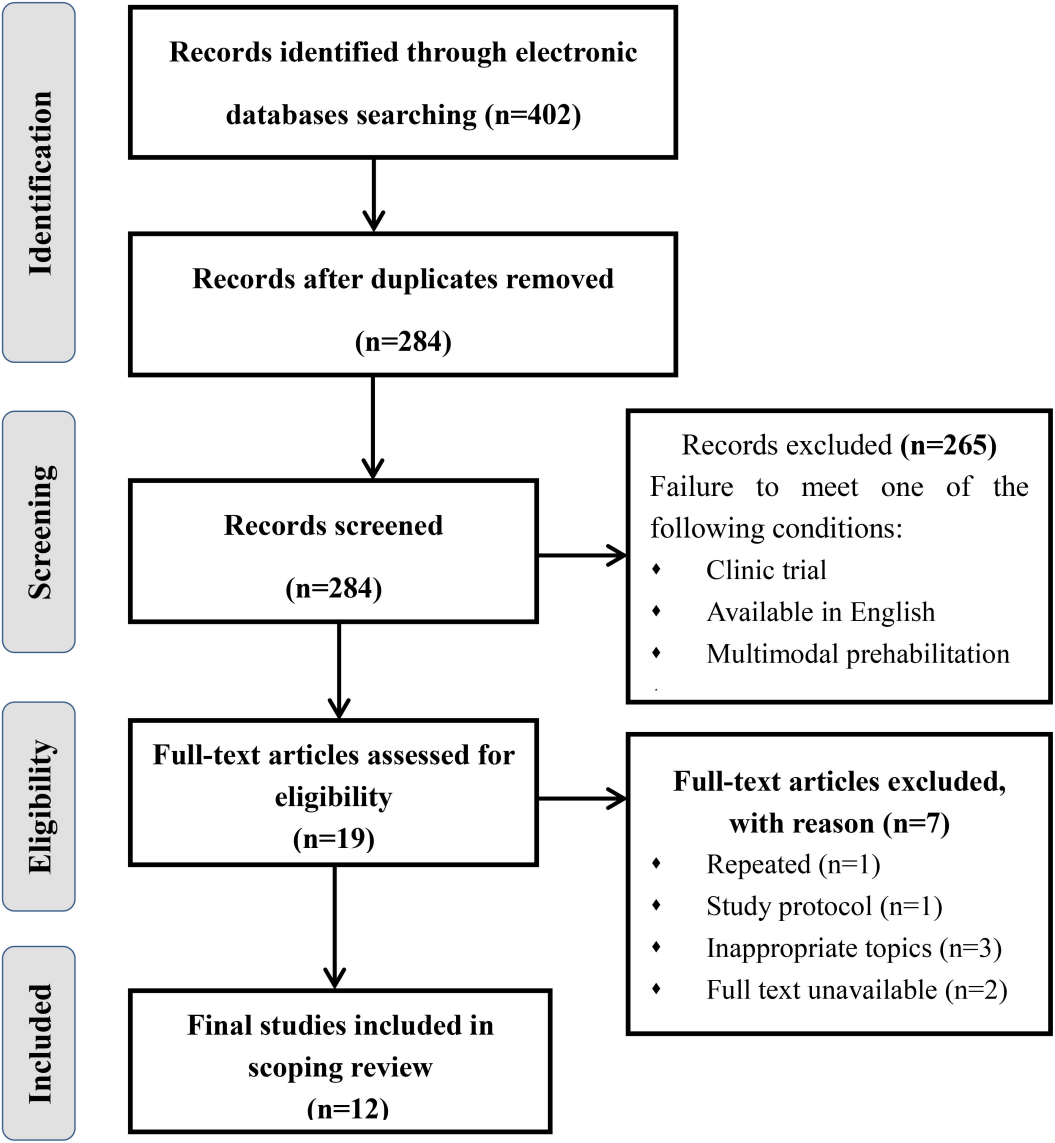
Preferred reporting for scoping reviews flow chart.

The initial search yielded a total of 402 documents. After removing duplicates, 284 papers remained. Of which 265 papers were excluded because their abstracts did not satisfy the inclusion criteria of “clinic trial”, “available in English”, and “multimodal prehabilitation”. After that, 19 articles were subjected to full-text review because the abstracts could not be ascertained to be eligible. Subsequently, 9 articles were omitted for the following reasons: repeated, study protocol, full text unavailable and inappropriate topics. Ultimately, a collection of 10 studies was included in this review. **Figure 1** displays the literature review process. All of them were published between 2017 and 2024. Most of the studies were originally from Canada (n = 4), Denmark (n = 1), the Netherlands (n = 2), Australia (n =1) or International multicenter (n=2). In addition, there are seven RCTs, a pilot intervention study, a cohort study and an emulated target trial design. **Table 2** shows the characteristics of the studies included in this review.

**Table 2:** Characteristics of the studies included in this review.

Author, Year	Country of Origin	Study design	Prehabilitation group	Control group	Intervention and Follow-up Duration	Supervision	Outcome measures
Molenaar et al, 2023 (11)	International multicenter	RCT	Mean age, years, mean (SD): 69 (12.59) Sex, female, n (%): 61 (49.6%) Sample size: 123	Mean age, years, mean (SD): 71 (11.85) Sex, female, n (%): 52 (40.6%) Sample size: 128	4 weeks, 8 weeks	Hospital supervision	Clavien-Dindo classification, 6-MWD; length of stay, readmissions, mortality, 1RM, handgrip strength, PG-SGA, peak VO_2_, VO_2_AT, GAD-7, PHQ-9, EORTC QLQ-C30
Carli et al, 2020 (12)	Canada	RCT	Mean age, years, mean (SD): 78 (7.41) Sex, female, n (%): 26 (47.3%) Sample size: 55	Mean age, years, mean (SD): 82 (6.67) Sex, female, n (%): 32 (58.2%) Sample size: 55	4 weeks, 4 weeks	Hospital and home-based supervision	Clavien-Dindo classification, length of stay, emergency department visits, readmissions, 6-MWD, 36-Item Short Form Survey, HADS, CHAMPS
Gillis et al, 2014 (13)	Canada	RCT	Mean age, years, mean (SD): 65.7 (13.6) Sex, female, n (%): 21 (55%) Sample size: 38	Mean age, years, mean (SD): 66.0 (9.1) Sex, female, n (%): 27 (69%) Sample size: 39	8 weeks, 8 weeks	Home-based supervision	6MWD, CHAMPS, compliance, readmission, length of hospital stay, 36-Item Short Form Survey, HADS, Clavien-Dindo classification, emergency department visits
Bojesen et al, 2023 (14)	Denmark	RCT	Mean age, years, mean (SD): 80(6.9) Sex, female, n (%): 5 (31%) Sample size: 16	Mean age, years, mean (SD): 78(6.3) Sex, female, n (%): 14 (70%) Sample size: 20	4 weeks, 3 days after surgery	Hospital supervision	QoR-15, 6-MWD, 30 s sit to stand test, 30 s stair climb test, hand grip strength, Hemoglobin level, PG-SGA, VO_2_, body weight, hemoglobin, creatinine, albumin, CCI, Clavien-Dindo classification
Bousquet-Dion et al, 2018 (15)	Canada	RCT	Mean age, years, mean (SD): 74 (7.78) Sex, female, n (%): 30 (81%) Sample size: 37	Mean age, years, mean (SD): 71(14.81) Sex, female, n (%): 16 (62%) Sample size: 26	4 weeks, 30 days after surgery	Home-based supervision	6MWD, length of stay, readmissions, emergency department visits, CHAMPS, inBody320V scale, grip strength, Clavien–Dindo classification, HADS
Waller et al, 2022 (16)	Britain	RCT	Mean age, years, mean (SD): 55.5 (9.26) Sex, female, n (%): 7 (63.6%) Sample size: 11	Mean age, years, mean (SD): 61.0 (11.70) Sex, female, n (%): 4 (36.4%) Sample size: 11	3 weeks, 1 day before surgery	Home-based supervision	6MWD, daily step counts, HADS, satisfaction, body weight, adverse events, compliance
Pesce et al, 2024 (17)	Italy	RCT	Mean age, years, mean (SD): 68 (8.7) Sex, female, n (%): 14 (41.7) Sample size: 35	Mean age, years, mean (SD): 70 (9.6) Sex, female, n (%): 15 (40.0) Sample size: 36	4 weeks, 8 weeks	Home-based Unsupervised	6MWD, VO2max, GAD-7, PHQ-9
Fulop et al, 2021 (18)	Hungary	RCT	Mean age, years, median (IQR [range]): 70 (60–75 (25–87)) Sex, female, n (%): 40 (51.9) Sample size: 77	Mean age, years, median (IQR [range]): 70 (64–75 (27–88)) Sex, female, n (%): 33 (45.8) Sample size: 72	3 to 6 weeks, 8 weeks	Home-based unsupervised and hospital supervision	6MWD, FVC, HADS, Clavien-Dindo classification, SF-36
Atoui et al, 2024 (19)	Canada	RCT	Mean age, years, mean (SD): 65.56 (12.63) Sex, female, n (%):18 (41.86) Sample size: 43	Mean age, years, mean (SD): 64.48 (14.41) Sex, female, n (%):25 (54.35) Sample size: 46	4 weeks, 8 weeks	Home-based unsupervised	CHAMPS, HADS, 6MWT, SF-36, PGSGA, Clavien-Dindo classification, Subjective sleep quality, Insomnia Severity Index, The World Health Organization Disability Assessment schedule
Groen et al, 2024 (20)	Netherlands	Cohort study	Mean age, years, mean (SD): 75.4 (6.6) Sex, female, n (%):26(53.1) Sample size: 49	Mean age, years, mean (SD): 75.5 (8.6) Sex, female, n (%):23(46.0) Sample size: 50	3 weeks, 90 days after surgery	Hospital supervision	Sit-to-stand test, 6MWD, steep ramp test, Clavien-Dindo classification, length of stay, readmission, mortality, Distress thermometer, 1RM, Short Nutritional Assessment Questionnaire, Charlson Co morbidity Index, Identification of the Seniors at Risk
Heil et al, 2023 (21)	Netherlands	Emulated target trial design	Mean age, years, mean (SD): 72 (5.9) Sex, female, n (%):55 (53.7) Sample size: 123	Mean age, years, mean (SD): 75 (6.7) Sex, female, n (%):53 (41.4) Sample size: 128	4 weeks, 4 weeks	Hospital supervision	Length of hospital stay, readmissions, comprehensive complication score
Suen et al, 2022 (22)	Australia	Pilot intervention study	Mean age, years, mean (SD): 72.5 (7.4) Sex, female, n (%):10 (45.4) Sample size: 50	NR	2–4 weeks, 4 weeks	Hospital supervision	6MWD, EORTC QLQ C30, PG SGASF, waist circumference, fat mass, fat-free mass, skeletal muscle mass, visceral adiposity, 30-second sit-to-stand, handgrip strength

RCT, randomized controlled trial; IG, intervention group; CG, control group; E, exercise; N, nutrition; P, psychology; 6-MWT, 6-minute walking distance; 1RM, 1 repetition maximum; PG-SGA, patient-generated subjective global assessment; GAD-7, Generalized Anxiety Disorder 7-item scale; PHQ-9, Patient Health Questionnaire 9-item; CHAMPS, Community Healthy Activities Model Program for Seniors; HADS, Hospital Anxiety and Depression Scale; PG SGASF, Patient-Generated Subjective Global Short-Form

### 3.1 Multimodal prehabilitation program

#### 3.1.1 Physical exercise intervention

The exercise interventions included in the study mainly included aerobic training, resistance training, breathing, balance and flexibility training. Intervention programs usually follow the “FITT” principle, which includes exercise frequency, exercise intensity, exercise duration, exercise type, and precautions (23). Common exercises include running, cycling, climbing stairs and swimming. When a program is implemented, patients are generally asked to mix aerobic exercise, anaerobic exercise and resistance exercise to allocate time reasonably (24).

#### 3.1.2 Nutritional intervention

This part of the intervention program focuses on instructing patients to take protein and vitamins according to existing guidelines (25).The guidelines recommended that surgical patients consume at least 1.2-2.0 g/kg of protein per day (26). The group of the European Society of Nutrition and Metabolism recommends that healthy adults consume at least 1.0-1.2 g/kg protein per day through a normal diet, and elderly people who are malnourished or at risk of malnutrition due to acute or chronic diseases should consume at least 1.2 - 1.5 g/kg of protein per day (27, 28). Moreover, 20–30 grams of whey protein are supplemented after exercise, and vitamins and minerals are supplemented if necessary to maximize the stimulation of the synthesis of muscle protein networks in healthy individuals (7).

#### 3.1.3 Psychological intervention

The psychological intervention included in the study generally began with the start of exercise and nutrition intervention, which generally uses relevant scales for initial assessment, and then, almost every weekly, psychological performed 1–2 times; each time, patients with psychological risk receive a higher level of intervention (8, 29). Psychological interventions commonly used in studies include teaching relaxation techniques (deep breathing, progressive muscle relaxation, and meditation), guided visualization (patients are asked to imagine the stages before and after surgery to control anxiety, or to imagine being in a safe, comfortable place to reduce stress), problem solving, and guidance on coping strategies.

#### 3.1.4 Others

In the interventions included in the present study, adverse lifestyle habits such as alcohol abuse and smoking were corrected before surgery, as these adverse health behaviors can affect patient prognosis and health-related outcomes (30, 31). Similarly, anemia increases the incidence of perioperative complications and mortality in patients and increases the likelihood of perioperative red blood cell transfusions. Patients with anemia are also targeted for intervention, with oral iron or even intravenous iron therapy if necessary (32). **Supplementary Appendix 2** showed the multimodal prehabilitation protocols for colorectal cancer.

### 3.2 Outcome measures

**Figure 2:**
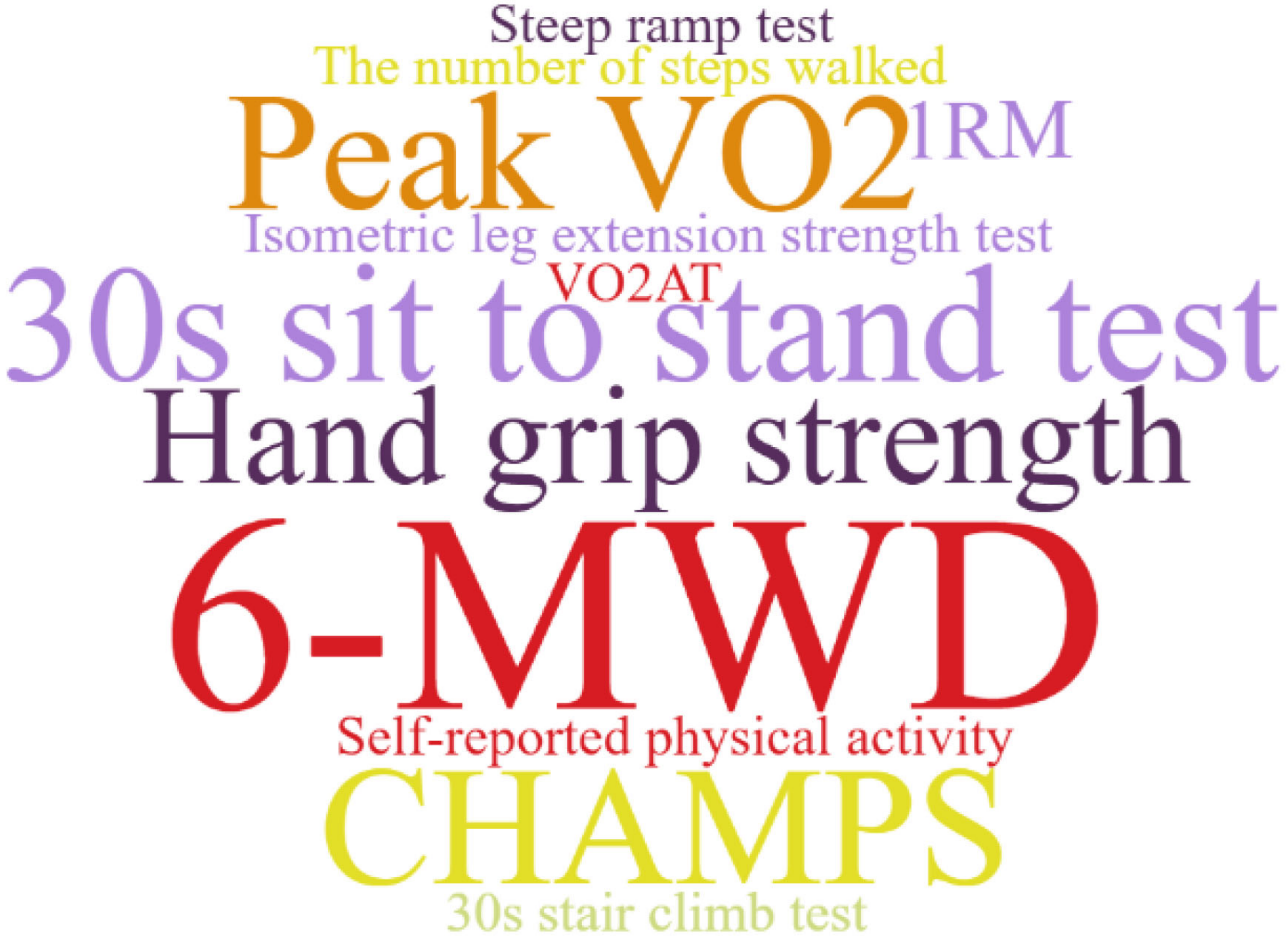
Functional capacity assessment word clouds.

**Figure 3:**
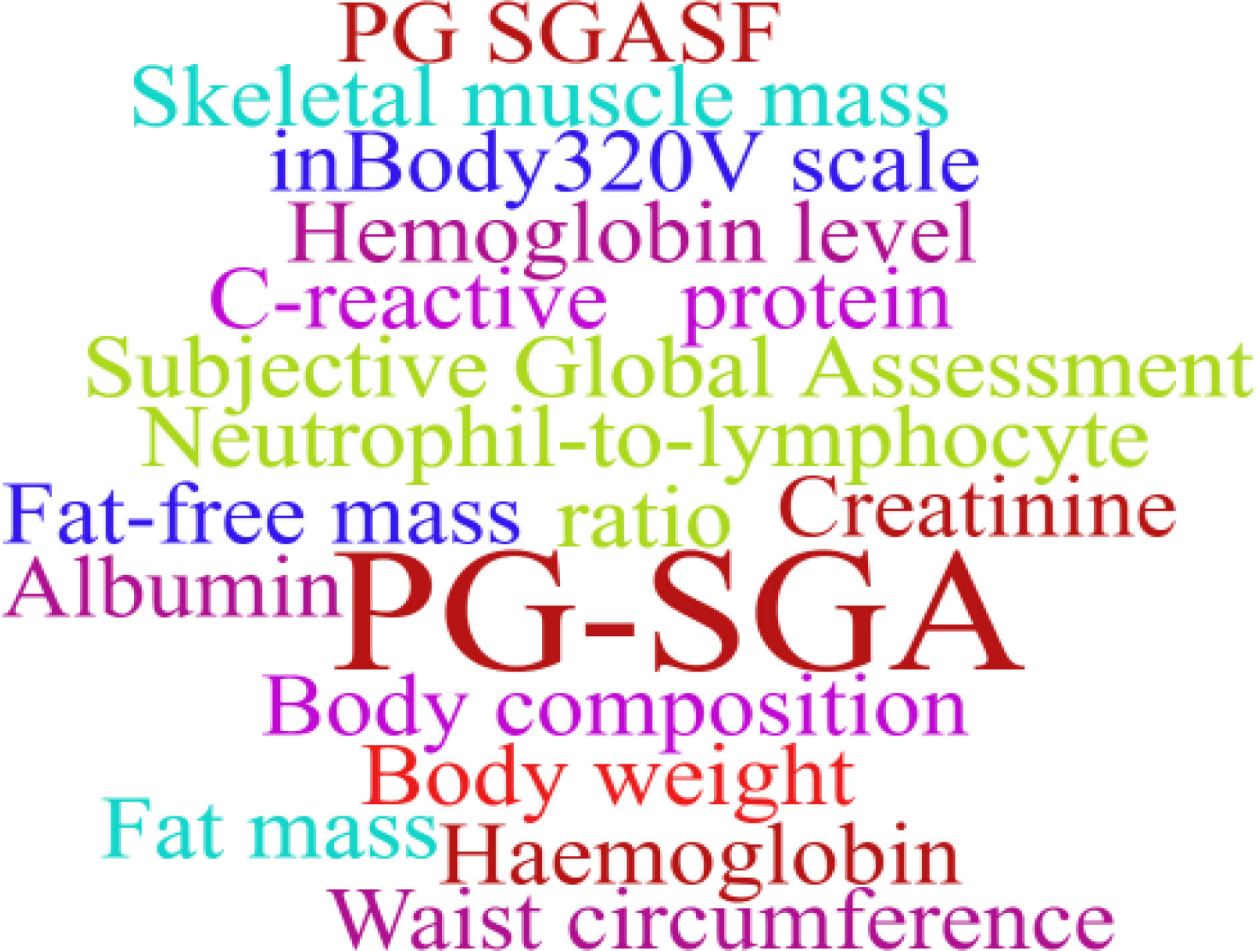
Nutritional assessment word clouds.

**Figure 4:**
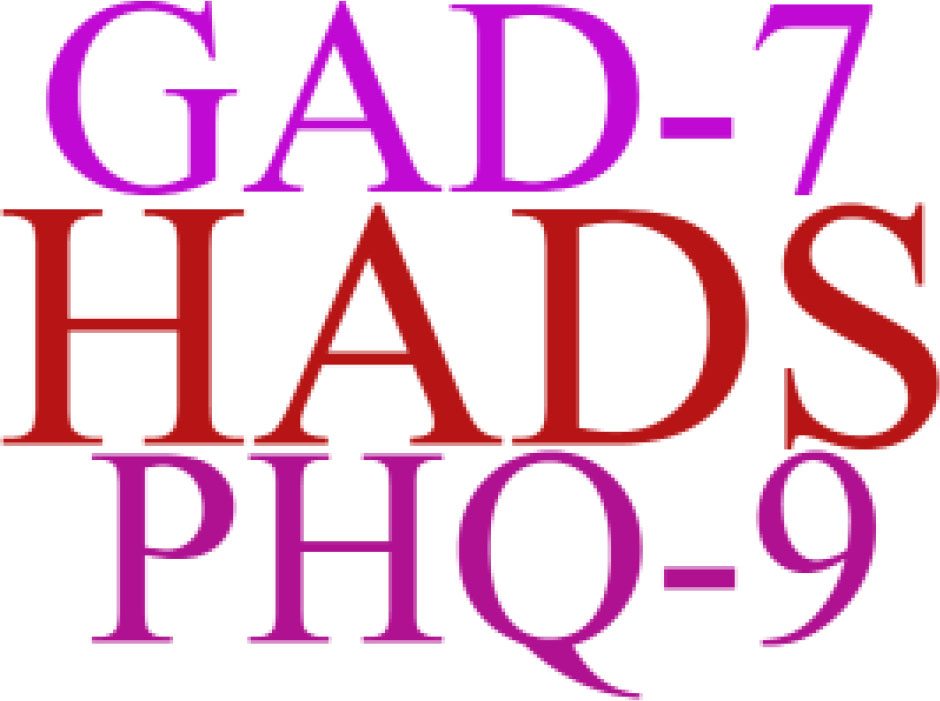
Psychological assessment word clouds.

**Figure 5:**
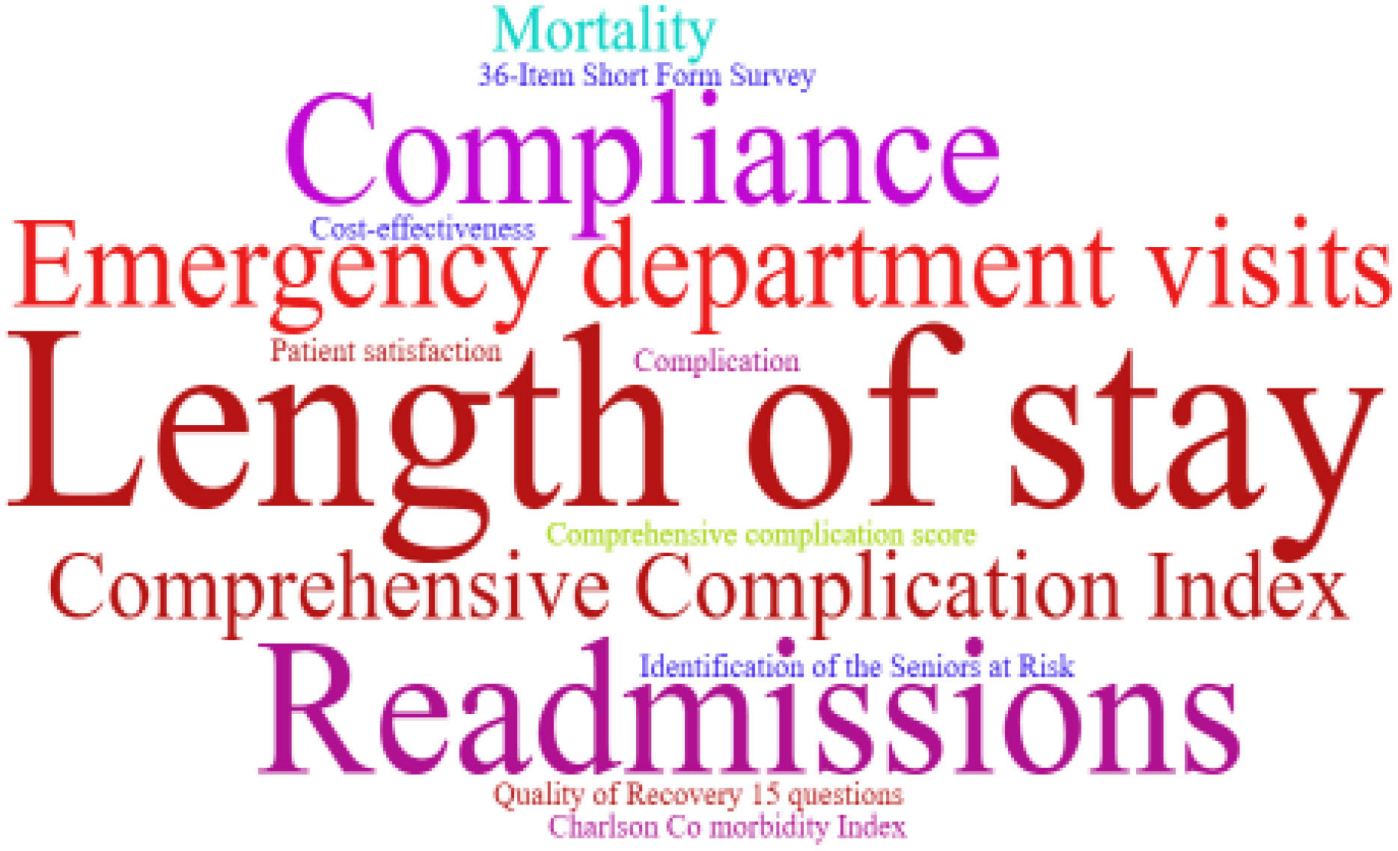
Surgical outcome correlation index word clouds.

**Figure 6:**
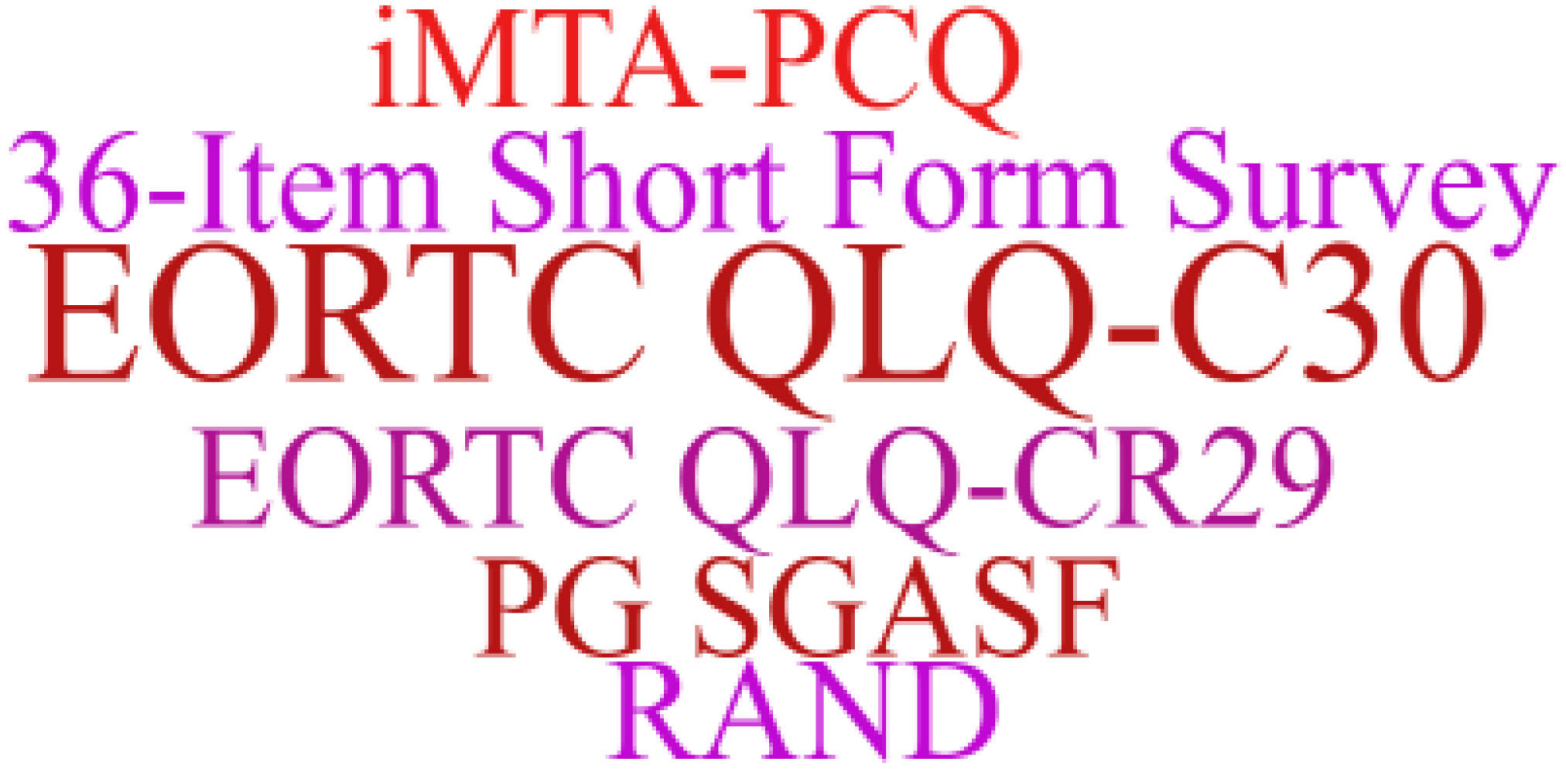
Health-related quality of life word clouds.

The use of prehabilitation measures before surgery in patients with CRC brings a range of benefits. We included the indicators in the article for statistics, and used a tool to represent the most frequent keywords in a larger font, and vice versa. As shown in **Figures 2**–**6**. The detailed frequency data are shown in **Supplementary Appendix 4**.

### 3.3 Duration, supervision, compliance and methods taken to improve compliance

#### 3.3.1 Duration and supervision

As shown in **Supplementary Appendix 3**, the duration in the studies ranged from 2–8 weeks, mostly 4 weeks. Patients receive prehabilitation at the hospital (11, 14), the community (20) and the home (13, 33), or in conjunction with the hospital and the home (12, 21, 22). The form of supervision can be divided into supervision and non-supervision. The choice depends on the patient and medical resources.

3.3.2 Compliance and methods taken to improve compliance

The compliance outcomes for prehabilitation among CRC patients reported in the included studies varied widely, between 50% and 100%, which may be related to intervention site, observation time, and observation content. Only three studies did not report methods to improve patients’ adherence to prehabilitation programs (11, 14, 21). The main methods used to improve compliance are telephone supervision and having patients report their progress regularly.

## 4 Discussion

The 10 studies included in this review described multimodal prehabilitation programs for patients undergoing elective CRC surgery, including exercise, nutrition and psychological intervention, which demonstrated that implementation of multimodal prehabilitation, has clinical benefits for CRC patients, such as reducing the incidence of postoperative complications and improving prognosis. However, due to the heterogeneity of multimodal prehabilitation programs in each study, this interferes with further standardized assessment of outcomes (34).

Initial preoperative rehabilitation is dominated by a single preoperative exercise intervention, typically consisting of aerobic, resistance and respiratory training aimed at improving patients’ preoperative functional reserve (35–37). Preoperative aerobic exercise is beneficial in reducing the risk of postoperative complications in patients undergoing major abdominal surgery, and patients with low fitness have a higher rate of postoperative complication morbidity and mortality, with increased cardiorespiratory-related complications being particularly pronounced (38, 39). Currently, the World Health Organization recommendations for the adult population are 150–300 min of moderate-intensity physical activity per week, 75–150 min of vigorous-intensity physical activity, or an equivalent combination of moderate-intensity and vigorous-intensity aerobic exercise (40). Exercise interventions should be presented in the form of an exercise prescription, which usually follows the “FITT” principle, i.e., it should include frequency, intensity, duration, type of exercise and precautions (23). However, the exercise interventions currently implemented do not satisfy the “FITT” principle, and the amount of exercise needs to be standardized, individualized for each patient, and captured and analyzed via lightweight e-health technology to facilitate the achievement of daily goals (41).

The role of nutritional interventions in the multimodal prehabilitation of CRC patients is not only to correct long-standing nutritional problems but also to alleviate the catabolic damage associated with the patient’s perioperative trauma (42). Additionally, adequate protein supplementation in an exercise intervention program is a goal of nutritional intervention, which facilitates muscle strength and improves functional reserve, improving clinical outcomes in patients with CRC (43). Patients who undergo major abdominal surgery are often malnourished because of reduced food intake and metabolic disruption caused by the cancer or tumor itself. Malnutrition, in turn, can lead to weakened physical function, which in turn increases complication rates, and readmission rates, prolongs hospitalization, and surgical recovery time, and decreases quality of life (44). The American Association for Accelerated Rehabilitation and the Perioperative Quality Advancement Consortium recommend that all patients undergoing elective abdominal surgery should be considered for preoperative immunonutritional intervention and that immunonutritional preparations should be administered 5 to 7 days before surgery (25). Nutritional interventions require periodic measurement of outcomes or indicators by the interventionist to assess the appropriateness of the nutritional prescription, so that adjustments can be made if the nutritional prescription does not adequately meet or exceeds the patient’s needs.

Psychological prehabilitation plays an important role in the preoperative multimodal prehabilitation of patients with CRC, alleviating preoperative anxiety while enhancing patients’ intrinsic drive for exercise and nutritional interventions, and improving adherence to regimens (45). Notably, although psychological interventions are recognized in the definition of the multimodal prehabilitation concept, this component is lacking in most current clinical intervention studies (46). Studies that have included psychological interventions in multimodal prehabilitation also suffer from inadequate descriptions, poor reporting of adherence, and substantial heterogeneity in duration and related outcome measures. In addition, with only a few studies risk-stratifying psychology, individualized coping strategies are missing, which contributes to heterogeneity (8).

There is heterogeneity in the evaluation indicators of the effects of multimodal prehabilitation between studies, which affects our further analysis, but also provides us with a direction of thinking. In the functional assessment of the included studies, the 6MWD was one of the most commonly used indicators. This index can simply reflect the basic functional reserve of the patient, the operation is simple, and it is also a method of exercise in the evaluation process. In addition, weekly aerobic and anaerobic exercise is an important part of multimodal prehabilitation, and the assessment of lung function should be increased, which is also the concept of pulmonary rehabilitation and may be associated with postoperative lung infection (47). The PG-SGA is relatively widely used in nutritional assessment, whereas the other indicators are related mainly to blood biochemistry and body composition measurements, however, these indicators have been used in only a few studies (14, 22). Future studies may use them to quantify nutrition-related outcomes, which is especially important for CRC patients.

Patient compliance in multimodal prehabilitation is a concern, especially in the unsupervised setting (48). In general, patients with CRC have 4 to 8 weeks of preoperative time to receive multimodal prehabilitation interventions at healthcare facilities, in the community, or at home. Prehabilitation is usually implemented under the supervision of healthcare professionals, and face-to-face and supervised forms of prehabilitation are considered the gold standard for implementing prehabilitation (49). Home-based prehabilitation is considered to have better implementation outcomes and higher levels of participation. The advantages of home-based prehabilitation programs include convenience and low cost, whereas the disadvantages include that compliance may be compromised for more severely ill patients and those who lack in-hospital supervision. The place of implementation depends on the patient’s preference and the availability of health resources, and regardless of the place of implementation of the program, it is necessary to assess patient adherence and provide reinforcement through regular feedback. With the advancement of technology and the widespread use of e-health technology in healthcare, patients can be supervised for home training through the use of tele-rehabilitation modalities such as video conferencing (16). Wearable devices can also be used to enable mutual understanding and feedback between patients and healthcare providers, which is a promising approach to improving adherence (50).

## 5 Conclusion

This review demonstrates the current evidence of multimodal prehabilitation in patients undergoing colorectal cancer surgery, showing a positive impact and beneficial trend on patient clinical outcomes. However, owing to the heterogeneity of multimodal prehabilitation in terms of specific content, duration, and evaluation metrics, many high-quality trials by researchers are still needed to explore the optimal model of multimodal prehabilitation and promote its standardization.

## Data Availability

The datasets presented in this study can be found in online repositories. The names of the repository/repositories and accession number(s) can be found in the article/**Supplementary Material**.
