# Supplementary material for: Multimodal prehabilitation program for patients undergoing elective surgery for colorectal cancer: a scoping review
Source: Front Oncol. 2025 Apr 30;15:1532624. doi: 10.3389/fonc.2025.1532624 (PMC12082037; doi:10.3389/fonc.2025.1532624)
Supplement: Supplementary file 1 [file Table1.docx]

Appendix 1. Search strategy for PubMed.

Appendix 2. Multimodal prehabilitation protocols for colorectal cancer.

Appendix 3. Compliance and ways to improve.

Appendix 4. Outcome measures.

**Appendix 1. Search strategy for PubMed.**

| **Search** | **Query** | **Results** |
| --- | --- | --- |
| **#1** | (((Pre-operative Exercise[Title/Abstract]) OR (Pre-operative Exercises[Title/Abstract])) OR (Preoperative Condition*[Title/Abstract])) OR (((((Prehabilit*[Title/Abstract]) OR (multimodal prehabilit*[Title/Abstract])) OR (Pre-habilit*[Title/Abstract])) OR (pre-operative rehab[Title/Abstract])) OR (preoperative rehab[Title/Abstract])) | 3333 |
| **#2** | **("Preoperative Exercise"[Mesh])** | 651 |
| **#3** | #1 OR #2 | 3389 |
| **#4** | **(Colorectal cancer [Title/Abstract])) OR (Colorectal Neoplasms [Title/Abstract])** | 145001 |
| **#5** | **("Colorectal Neoplasms"[Mesh])** | 249008 |
| **#6** | #4 OR #5 | 291447 |
| **#7** | #3 AND #6 | 244 |

**Appendix 2. Multimodal prehabilitation protocols for colorectal cancer.**

| **Author, Year** | **Exercise** | **Nutritional intervention** | **Mental intervention** | **Others** |
| --- | --- | --- | --- | --- |
| Molenaar et al, 2023 ^[11]^ | 1-hour session of aerobic and strength exercises 3 times per week | A daily amount of proteins of 1.5 g per kg; ingest 30 g within 1 hour after the session and 1 hour before sleeping daily. Vitamin D and multivitamin supplements were also provided | Anxiety-coping interventions provided by psychology-trained personnel in a 1-to-1 session | Smoking cessation |
| Carli et al, 2020 ^[12]^ | 30 minutes of moderate aerobic exercise, 25 minutes of resistance exercises and 5 minutes of stretching. A personalized home-based program of aerobic activities | Target protein intake was 1.5 g/kg of body weight, and provide whey protein supplements if necessary | Individualized psychological coping strategies | Smoking and alcohol cessation |
| Gillis et al, 2014 ^[13]^ | 50 minutes of aerobic and resistance training at least 3 times a week | 3 days of individualized dietary instruction at the beginning; 1.2 g of protein per kilogram of body weight | A psychologist instructs anxiety reduction techniques two to three times a week | NR |
| Bojesen et al, 2023 ^[14]^ | Supervised high-intensity and low-intensity interval training three times a week, followed by resistance training for large muscle groups | Within the first week the dietitian makes personalized recommendations and then uses a protein supplement prescription (30 g of protein twice a day), vitamins and multivitamins | General Anxiety Disorder Global Assessment, Patient Health Questionnaire | NR |
| Bousquet-Dion et al, 2018 ^[15]^ | Do 30 minutes of moderate-intensity aerobic exercise three to four times a week and resistance training three to four times a week | To achieve a total protein intake of 1.2 g/kg/ day, take protein and/or supplements within an hour of exercise training | Personalized technology to ease anxiety | NR |
| Waller et al, 2022^[16]^ | It's personalized and structured  exercise and physical activity programme, aerobic exercise (3×per week), resistance exercise using a resistance band consisting of 8–10 repetitions in two sets (2×per week) | Use the Fitbit app's food log to monitor your daily dietary intake and increase your protein intake | Complete a guided meditation each day using a mindfulness app that offers stress management and relaxation techniques | NR |
| Pesce et al, 2024^[17]^ | 3 sessions per week interval and resistance training (walking or cycling) | A daily amount of proteins of 1.5 g per kg, 30 mg of whey protein 1 h after physical exercise and 1 h before bed time, multivitamins and omega-3 fatty acid as extra supplements | If indicated, 90-min psychological intervention in the first session and additional sessions during the 4-week prehabilitation period | NR |
| Fulop et al, 2021^[18]^ | Thirty minutes of daily moderate intensity aerobic activity (walking or jogging according to patient abilities) was recommended. In addition, deep breathing/coughing exercises (10–15 min daily) and incentive spirometer exercises were suggested (four or five times a day) | Patients were provided with oral nutritional supplementation when necessary based on the ESPEN guidelines. Patients were asked to record the consumption of prescribed oral nutritional supplementation units | Each patient received a 60-min session under the supervision of a trained psychologist, who provided personalized techniques for reducing anxiety | Smoking cessation and alcohol abstinence |
| Atoui et al, 2024^[19]^ | up to 50 min of exercise for at least three days per week, alternating between aerobic and resistance training, Each session included a 5-min warm-up, 20 min of aerobic exercise, 20 min of resistance training, and a 5-min cool down | Provide personalized dietary guidance based on ESPEN guidelines to calculate dietary protein and energy intake | participants who scored more than 6 points in the HADS-Anxiety or more than 8 in the HADS-Depression received up to a 60-min visit with a trained psychologist who provided techniques to reduce anxiety | NR |
| Groen et al, 2024 ^[20]^ | High-intensity interval and strength training three times a week | Aim for at least 25-30 grams of protein and a multivitamin supplement at each meal | Mental distress was assessed with the Distress thermometer and participants practice relaxation exercises with the physical therapist | Quit smoking  Smoking and alcohol cessation, screening and treatment of frailty and anemia |
| Heil et al, 2023 ^[21]^ | 60 minutes of high-intensity training three times a week, and 60 minutes of low-intensity endurance training four times a week | Tailored nutritional recommendations to achieve a total protein intake of 1.9 grams per kilogram of lean body mass per day, supplemented with additional protein when necessary | Nurses and psychologists provide psychological support | Cure anemia, quit smoking and drinking |
| Suen et al, 2022 ^[22]^ | Participate in 60 minutes of supervised aerobic and resistance exercise twice a week | Personalize your protein intake according to the guidelines | Twice weekly by a CRC nurse specialist to provide general support and encouragement, assess patient engagement and resolve problems in a timely manner | NR |

NR, not reported

**Appendix 3.** **Compliance and ways to improve.**

| **Author, Year** | **Compliance to Program** | **Ways to improve** |
| --- | --- | --- |
| Molenaar et al, 2023 ^[11]^ | At least 75% | NR |
| Carli et al, 2020 ^[12]^ | Mean (SD)  In-hospital: 68% (38%)  Self-reported: 80% (27%) | Patients use a standardized instruction manual to record daily activities  Patients report weekly by phone on their compliance with the home component of the program |
| Gillis et al, 2014 ^[13]^ | Compliance during preoperative period, % (SD)= 78 (21)  Compliance from surgery to 4 weeks, % (SD)= 53 (30)  Compliance from 4 to 8 weeks, % (SD)= 53 (33) | Patients were contacted weekly by telephone and assessed with a standardized set of open-ended questions to uncover issues related to maintaining compliance |
| Bojesen et al, 2023 ^[14]^ | Training intervention, median: both group 100% (range: 80–100% and 64–100%, respectively)  Nutritional supplements, median: 98% (range: 0–100%) | NR |
| Bousquet-Dion et al, 2018 ^[15]^ | Exercise: Prehab, Control (%)  Preop: 98; 4 weeks: 72, 79 ;8 weeks:82, 75  Nutrition: Prehab, Control (%)  Preop: 100; 4 weeks: 91, 84 ;8 weeks:92, 83 | NR |
| Waller et al, 2022^[16]^ | Exercise  prehabilitation group: 59.9% (95% CI, 41.3–78.5)  control group: 42.6% (95% CI 18.4–66.7)  Nutrition  prehabilitation group, median: 82.9%  Psychosocial support: 15% | Use apps as motivational tools for exercise and nutrition, Standardized structured calls are provided weekly |
| Pesce et al, 2024^[17]^ | About 80% | NR |
| Fulop et al, 2021^[18]^ | NR | Patients are asked to record all activities in a booklet containing a multimodal prehabilitation program |
| Atoui et al, 2024^[19]^ | Participants in the prehab group reported commitment to the exercise prescription of 85.6% (18.69) and 96.9% (7.78) adherence to the nutrition program | Require patients to record all activities of recovery; Patients are contacted weekly by phone and assessed through a standardized set of open-ended questions |
| Suen et al, 2022 ^[22]^ | Supervised exercise: 78.5% (range 33.3-100%)  Nurse-led support sessions: 65.6% (range 0-100%) | Phone support and encouragement. Assess patient status to determine if there are any additional concerns and encourage adherence to the intervention |
| Groen et al ^[20]^ | 98.0% | Patients were asked to keep a journal of nutritional supplements and low-impact training. One week after the start of prehabilitation, the dietician checked the diet and adherence by telephone |
| Heil et al ^[21]^ | NR | NR |

NR, not reported

**Appendix 4. Outcome measures**

3.2.1. Functional capacity assessment

| Item | Frequency |
| --- | --- |
| 6-MWD | 9 |
| VO_2_AT | 1 |
| Peak VO_2_ | 3 |
| 1RM | 3 |
| Hand grip strength | 6 |
| Self-reported physical activity | 1 |
| Isometric leg extension strength test | 1 |
| 30s stair climb test | 1 |
| 30s sit to stand test | 3 |
| Steep ramp test | 1 |
| The number of steps walked | 1 |
| CHAMPS | 3 |

3.2.2. Nutritional assessment

| Item | Frequency |
| --- | --- |
| PG-SGA | 3 |
| Body weight | 1 |
| Short Nutritional Assessment Questionnaire | 1 |
| Hemoglobin level | 1 |
| Haemoglobin | 1 |
| Neutrophil-to-lymphocyte ratio | 1 |
| C-reactive protein | 1 |
| Albumin | 1 |
| Body composition | 1 |
| Creatinine | 1 |
| Nutritional Risk Screening tool NRS2002 | 1 |
| inBody320V scale | 1 |
| Subjective Global Assessment | 1 |
| PG SGASF | 1 |
| Waist circumference | 1 |
| Fat mass | 1 |
| Fat-free mass | 1 |
| Skeletal muscle mass | 1 |

3.2.3. Psychological assessment

| Item | Frequency |
| --- | --- |
| GAD-7 | 2 |
| PHQ-9 | 2 |
| HADS | 3 |

3.2.4. Surgical outcome correlation index

| Item | Frequency |
| --- | --- |
| Length of stay | 8 |
| Readmissions | 6 |
| Mortality | 2 |
| Emergency department visits | 4 |
| Compliance | 7 |
| 36-Item Short Form Survey | 1 |
| Clavien-Dindo classification | 7 |
| Comprehensive Complication Index | 3 |
| Quality of Recovery 15 questions | 1 |
| Charlson Co morbidity Index | 1 |
| Identification of the Seniors at Risk | 1 |
| Comprehensive complication score | 1 |
| Patient satisfaction | 1 |
| Cost-effectiveness | 1 |
| Complication | 1 |

3.2.5. Health-related Quality of Life

| Item | Frequency |
| --- | --- |
| 36-Item Short Form Survey | 1 |
| EORTC QLQ-CR29 | 1 |
| EORTC QLQ-C30 | 3 |
| RAND | 1 |
| iMTA-PCQ | 1 |
| PG SGASF | 1 |
